# Predictors of vascular complications among type 2 diabetes mellitus patients at University of Gondar Referral Hospital: a retrospective follow-up study

**DOI:** 10.1186/s12902-018-0280-0

**Published:** 2018-07-31

**Authors:** Haileab Fekadu Wolde, Asrat Atsedeweyen, Addisu Jember, Tadesse Awoke, Malede Mequanent, Adino Tesfahun Tsegaye, Shitaye Alemu

**Affiliations:** 1 Department of Epidemiology and Biostatistics, Institute of Public Health, College of Medicine and Health Sciences, University of Gondar, Gondar, Ethiopia; 2 Department of Internal Medicine, School of Medicine, College of Medicine and Health Sciences, University of Gondar, Gondar, Ethiopia

**Keywords:** Incidence, Predictor, Type 2 diabetes

## Abstract

**Background:**

Type 2 Diabetes Mellitus is a serious metabolic disease that is often associated with vascular complications. There are 1.9 million people living with Diabetes in Ethiopia; diabetes mellitus is found to be the ninth leading cause of death related to its complications. Although the rate of vascular complications continues to rise, there is limited information about the problem. This study aimed to estimate the incidence and predictors of vascular complications among type 2 diabetes mellitus patients at University of Gondar Referral Hospital.

**Methods:**

Institution based retrospective follow-up study was conducted at University of Gondar Referral Hospital with 341 newly diagnosed type 2 DM patients from September 2005 to March 2017 and the data were collected by reviewing their records. Schoenfeld residuals test and interaction of each covariate with time were used to check proportional hazard assumption. The best model was selected by using Akaike Information Criteria (AIC). Hazards ratio (HR) with its respective 95% confidence interval were reported to show strength of association.

**Result:**

The selected patients were followed retrospectively for a median follow up time of 81.50 months (Inter quartile range (IQR) = 67.2–103.3). The mean age (± Standard deviation (SD)) of patients at baseline was 51.7(SD: ±11.5 years) and 57.48% were females. The incidence rate of vascular complications was 40.6 cases/ 1000 person years of observation. The significant predictors for vascular complications where found to be male sex (Adjusted hazard ratio (AHR) = 0.50, 95% CI: 0.27, 0.94), having hypertension at baseline(AHR = 3.99, 95% CI: 1.87, 8.56), positive protein urea at base line (AHR = 1.69, 95% CI: 1.03, 2.78), high density lipoprotein cholesterol(HDL-C) level ≥ 40 mg per deciliter (mg/dl) (AHR = 0.43, 95% CI: 0.24, 0.77), low density lipoprotein cholesterol(LDL-C) level > 100 mg/dl (AHR = 3.05, 95% CI: 1.47, 6.35) and triglyceride > 150 mg/dl (AHR = 2.74, 95% CI: 1.28, 5.84).

**Conclusion:**

The incidence of vascular complications among type 2 diabetes patients remains a significant public health problem. Hypertension at baseline, LDL-C > 100 mg/dl, triglyceride > 150 mg/dl, HDL-C ≥ 40 mg/dl and male sex were significant predictors of vascular complication. In the light of these findings targeted interventions should be given to diabetes patients with hypertension comorbidity and dyslipidemia at follow up clinics.

## Background

Diabetes mellitus(DM) is a chronic metabolic disorder characterized by chronic hyperglycemia [1]. Globally, the prevalence of DM is 8.5% and it is estimated that one in 10 adults will have DM in the world by 2035 [2]. Sub Saharan African countries are expected to experience the fastest increase in the number of people living with type 2 DM in the next two decades worldwide [3]. Ethiopia is the third most populous country in the African continent with 1.9 million people living with DM [4].

The seriousness of DM is largely a result of its associated vascular complications, which can be disabling and even fatal. Vascular complications caused by type 2 DM include neuropathy, nephropathy, retinopathy, coronary heart diseases(CHD), peripheral arterial diseases (PAD) and stroke [5].

In Africa, the age standardized mortality rate due to DM and its complications is estimated to be 111.3 per 100,000 population [2]. The estimated prevalence of diabetic nephropathy is 6–16% in Sub Saharan Africa [6] and 6.1% in Ethiopia [7].In addition,the prevalence of retinopathy ranges from 31.4–41.1% in Ethiopia [8]. Type 2 DM is rapidly increasing non-communicable disease and is a major public health challenge in developing countries like Ethiopia [9] with consequences of chronicity and pre-mature death due to its vascular complications [10, 11]. In Ethiopia there were 44,655 deaths between 2012 and 2013 among people aged 20–79 years due to DM and its associated vascular complications. It was the ninth leading cause of death in Ethiopia with 22 per 1000 deaths [3, 4, 12].

There are factors which can affect the rate of vascular complications among type 2 DM patients. Among socio demographic variables females experienced higher rates of vascular complication when compared to males as the studies done in Ethiopia and India [13, 14] but not in other studies [15, 16]. Individuals who were hypertensive at the start of treatment have a positive association with the risk of vascular complications [15–19]. Patients with higher level of LDL-C and lower levels of HDL-C were at increased risk of developing vascular complications [20–22]. Higher levels of cholesterol and triglyceride were also positively associated with the risk of vascular complications [15].

Ethiopia is facing a double burden problem because type 2 DM is currently increasing due to different factors such as aging, urbanization, and an increasing prevalence of obesity. Even though the rate type 2 DM and its associated vascular complications are rising, current updated information about the problem is limited. The available literatures indicated that there were discrepancies in findings for some variables, like sex. Therefore, it is imperative to conduct a study which assesses the association between incidence of vascular complications against socio-demographic, clinical and physiologic factors using a parametric survival model.

Identifying factors which influence the rate of vascular complications would provide information for health professionals, policy makers and other governmental and non-governmental organizations to maximize efforts on prevention and risk minimization of vascular complications and deaths due to the complications in the country as well as in the study area. Thus this study aimed to determine the incidence and predictors of vascular complications among type 2 DM patients in University of Gondar Referral Hospital, Ethiopia.

## Methods

### Study design and period

Institution based retrospective follow up study was conducted among type 2 DM patients at University of Gondar Referral Hospital. Newly diagnosed type 2 DM patients who were enrolled between September 2005 and August 2012 were followed up to March 2017.

### Study area and population

University of Gondar Referral Hospital is located in the North Gondar administrative zone, Amhara National Regional State, which is about 750 k meters (KM).

Northwest of Addis Ababa. The University of Gondar Referral Hospital is a teaching hospital which serves more than five million people in the North Gondar zone and its neighboring zones. Around 24,552 patients have chronic disease follow-up per year and among these 8880 are DM patients..Among all type 2 DM patients who are newly diagnosed between September 2005 and August 2012, newly diagnosed patients (364) who were free from any of the vascular complications at the start of treatment were selected randomly and included to the study. Patients with missing key predictor variables at baseline such as: HDL-C, LDL-C, triglyceride and hypertension status were excluded from the study.

### Data collection procedures and data quality control

The study used secondary data; a data extraction check list was prepared to collect the data. Type 2 DM patients who were newly diagnosed between September 2005 and August 2012 were included. However patients who had any vascular complications mentioned at the start of the study and patients who were missing the key variables were excluded from the study. The reviewed records were identified by their medical registration or card number. The primary outcome was having any of the vascular complications such as: retinopathy, nephropathy, neuropathy, stroke, peripheral arterial dieses and coronary heart disease. These complications were determined based on the clinical decision of the physician. Diabetic retinopathy was defined by both direct and indirect ophthalmoscopy assessments done by retinal specialists confirmed by fundus photography. Neuropathy was defined by history of numbness, paraesthesia, tingling sensation confirmed by touch sensation by 10 g monofilament, vibration sense by biothesiometer and ankle reflex. Nephropathy was defined as worsening of blood pressure control, swelling of feet ankle, hands or eyes, increased need to urinate, protein in the urine with a confirmation by tests like blood test, urine test, renal function test and imaging test. Stroke is defined as patients with sudden difficulty in speech and comprehension, sudden paralysis or numbness of the face, arm or leg, sudden trouble with walking and confirmation imaging with computerized tomography (CT) scan or magnetic resonance imaging (MRI). PAD was defined by history of intermittent claudication**,** coldness in the lower extremities (especially when compared with the other side), weak or absent peripheral pulses in the lower extremities and confirmation via Doppler ultrasound. CHD was diagnosed by symptoms of angina, shortness of breath, a crushing sensation in the chest, pain in the shoulder or arm and sweating. Additionally CHD was confirmed by electrocardiogram (ECG) or echocardiogram [23, 24]. The patients who were included in the study were assessed for all of these vascular complications in every follow up they had in the hospital. All baseline characteristics at the start of treatment were assessed from the patient’s registration document. The first characteristic assessed was the socio demographic component; this included age, sex and residence. The second characteristic assessed was the clinical component; this included hypertension comorbidity which was defined as a history of antihypertensive drug use or SBP ≥ 140 mmHg or DBP ≥ 90 mmHg [25], type of treatment, family history of DM, and body mass index (BMI). The third characteristic assessed was the physiologic component; this included HDL-C, LDL-C, triglyceride and total cholesterol which were categorized as high and low based on guidelines from the National Cholesterol Education Program (NCEP-III) and World Health Organization (WHO) [26, 27]. This also included creatinine, fasting blood sugar, systolic blood pressure(SBP), diastolic blood pressure(DBP) and protein urea which was defined as positive if the urine albumin concentration is between 30 mg(mg)/24 h and 300 mg/ 24 h and negative if it is < 30 mg/24 h. All of these characteristics of the patients were collected from their registration document. The data was collected by two health officers who had experience working in DM follow-up clinics. To control the data quality, training was given to the data collectors and their supervisor. The data extraction checklist was pre-tested for consistency of understanding the review tools and completeness of data items. The necessary adjustments were made on the final data extraction format and the filled formats were checked daily by the supervisor.

### Data management and analysis strategy

The data was entered in to EPI info version 7.0 and transferred to STATA version 14.1 for analysis. Descriptive statistics were used to describe the percentage and frequency of the patients in reference to all covariates. Person-time at risk was measured starting from the time of initiation of treatment until each patient ended the follow-up. The survival experience of the patients was assessed using Kaplan-Meier survivor function. The log rank test was used to compare the survival experiences among the different groups of subjects. Schoenfeld residuals test (both global and scaled), interaction of each covariate with time and graphical methods were used to check the Cox Proportional Hazard (PH) assumption. Cox PH and three parametric models (Exponential, Weibull and Gompertz) models were fitted to identify the risk factors. The best model was selected by using Akaike information criteria (AIC), Bayesian information criteria (BIC) and log likelihood criteria. Goodness of fit of the model was assessed by using cox-snell residual technique. Variables having p - value less than 0.05 in the multivariable model were considered significantly associated with the dependent variable. Hazard ratio (HR) with its 95% confidence interval were computed to show the strength of association.

## Result

### Baseline characteristics of study participants

Out of the total of 341 newly diagnosed type 2 DM patients, 196 (57.48%) were females. The mean (SD) age for patients at the start of treatment was 51.7 (SD ± 11.5) years. The majority of the patients 273(80.06%) were urban dwellers. About 228(66.86%) of the patients had family history of DM and more than half of the patients 183(53.67%) had hypertension at the start of type 2 DM treatment. Almost half of the study participants 169(49.56%) had normal weight whereas 45(13.2%) were obese. About 230(67.45%) were on oral hypoglycemic agents. The majority of the patients 271(79.47%) had positive protein urea at base line. About 234(68.62%) of the patients had HDL-C level above 40 mg/dl and more than half of the patients 186(54.55%) had LDL-C level less than 100 mg/dl. More than half of type 2 DM patients included in the study 178(52.2%) had triglyceride level ≤ 150 mg/dl. The median value for creatinine and FBS was found to be 0.78 mg/dl (IQR = 0.65–0.88) and 77 mg/dl (IQR = 121–178) respectively. The mean (±SD) for SBP and DBP of the patients was 126.9(±15.8) and 78.9 (±10.1) respectively (Table 1).

**Table 1:** Socio-demographic, clinical and physiologic characteristics of type 2 DM patients on anti diabetic treatment at university of Gondar referral hospital, September, 2005 – March 2017

Variable	No Vascular complication (*n* = 244)	Any one of vascular complications (*n* = 97)	Total (*n* = 341)
Sex			
Female *n(%)*	128(52.5)	68(70.1)	196(57.5)
Male *n(%)*	116(47.5)	29(29.9)	145(42.5)
Residence			
Rural *n(%)*	54(22.1)	14(14.4)	68(19.9)
Urban *n(%)*	190(77.9)	83(85.6)	273(80.1)
Occupation			
Unemployed *n(%)*	92(37.7)	57(58.8)	149(43.7)
Government *n(%)*	71(29.1)	17(17.5)	88(25.8)
NGO *n(%)*	16(6.6)	4(4.1)	20(5.9)
Private *n(%)*	65(26.6)	19(19.6)	84(24.6)
Age(year)^a^	50.1 ± 11.7	55.9 ± 9.9	51.7 ± 11.5
Family history			
Yes *n(%)*	178(73.0)	50(51.5)	228(66.9)
No *n(%)*	66(27.0)	47(48.5)	113(33.1)
Hypertension			
No *n(%)*	148(60.7)	10(10.3)	158(46.3)
Yes *n(%)*	96(39.3)	87(89.7)	183(53.7)
BMI (kg/m^2^)			
< 18.5 *n(%)*	24(9.8)	5(5.2)	29(8.5)
18.5–24.99 *n(%)*	137(56.2)	32(33.0)	169(49.6)
25–29.99 *n(%)*	62(25.4)	36(37.1)	98(28.7)
≥ 30 *n(%)*	21(8.6)	24(24.7)	45(13.2)
Treatment			
OHA *n(%)*	163(66.8)	67(69.1)	230(67.4)
Insulin *n(%)*	48(19.7)	16(16.5)	64(18.8)
OHA + Insulin *n(%)*	33(13.5)	14(14.4)	47(13.8)
Protein urea			
Negative *n(%)*	217(89.0)	54(55.7)	271(79.5)
Positive *n(%)*	27(11)	43(44.3)	70(20.5)
HDL-C(mg/dl)			
< 40 *n(%)*	36(14.8)	71(73.2)	107(31.4)
≥ 40 *n(%)*	208(85.2)	26(26.8)	234(68.6)
LDL-C(mg/dl)			
≤ 100 *n(%)*	174(71.3)	12(12.4)	186(54.5)
> 100 *n(%)*	70(28.7)	85(87.6)	155(45.5)
Triglyceride(mg/dl)			
≤ 150 *n(%)*	165(67.6)	13(13.4)	178(52.2)
> 150 *n(%)*	79(32.4)	84(86.6)	163(47.8)
Cholesterol(mg/dl)			
≤ 200 *n(%)*	184(75.4)	27(27.8)	211(61.9)
> 200 *n(%)*	60(24.6)	70(72.2)	130(38.1)
FBS (mg/dl)^b^	136(117.5–165.5)	200(152–249)	146(121–198)
Creatinine(mg/dl)^b^	0.76(0.63–0.84)	0.83(0.68–1.13)	0.78(0.65–0.88)
SBP(mm Hg)^a^	122.9 ± 14.0	137.0 ± 15.8	126.9 ± 15.8
DBP(mm Hg)^a^	76.7 ± 9.3	84.5 ± 10.0	78.9 ± 10.1

^a^Expressed as mean ± SD and ^b^median inter quartile range. *BMI* body mass index, *DBP* diastolic blood pressure, *FBS* fasting blood sugar, *HDL-C* high density lipoprotein cholesterol, *LDL-C* low density lipoprotein cholesterol, *SBP* systolic blood pressure

### Vascular complications from type 2 DM

Study subjects were followed for a median (IQR) follow up period of 81.5 months (IQR = 67.2–103.3) after initiation of treatment for a total of 2391.067 person years. During this time period the incidence of vascular complications was found to be 40.6 cases (95% CI: 33.2, 49.5) per 1000 person year observation. From this the incidence of retinopathy was 18.4 (95% CI: 8.8, 38.6), nephropathy was 14.4(95% CI: 9.8, 21.4), neuropathy was 18.9(95%CI: 13.7, 25.9), stroke was 17.0(95%CI: 8.5, 33.9), CHD was 16.7(95%CI: 8.7, 32.1) and PAD was 15.1(95%CI: 7.9, 29.0) cases per 100 person year of observation.

**Fig. 1:**
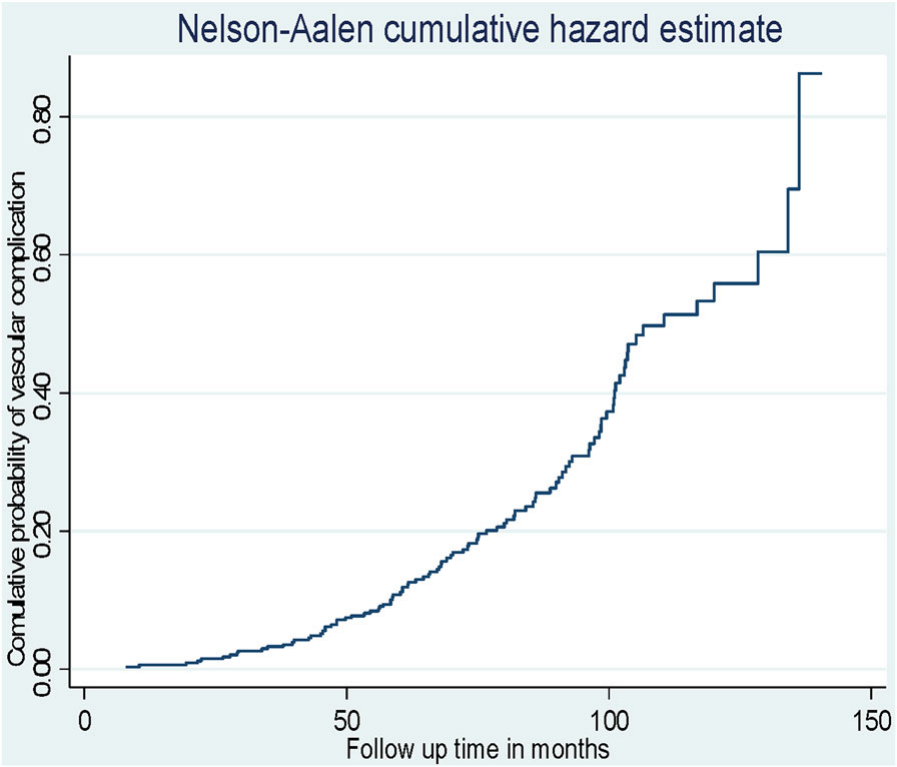
The Nelson-Aalen estimated cumulative curve showing cumulative probability of vascular complications among type 2 DM patients on anti-diabetic treatment at University of Gondar Referral Hospital, September, 2005 – March, 2017

The cumulative probability of developing vascular complications among type 2 DM patients who were free from any of the complications at the start of treatment was 0.0423 at month 40, 0.1653 at month 70, 0.3726 at month 100, 0.5587 at month 120 and 0.8617 at month 140 during the follow up period (Fig. 1).

### Predictors of vascular complication among type 2 DM patients

After multivariable analysis using the Gompertz Cox-Regression: covariates like sex, hypertension status at baseline, protein urea at baseline, HDL-C level, LDL-C, triglyceride level were found to be independent predictors for vascular complications among type 2 DM patients (Table 2). The risk of developing vascular complications is decreased by 50% among male type 2 DM patients than female patients. Positive protein urea at the start of treatment increased the risk of vascular complications by 69% as compared to negative protein urea. The risk of vascular complications for patients who have hypertension at baseline was 3.99 times higher than that of patients who have no hypertension. HDL-C level ≥ 40 mg/dl at the start of anti-diabetic treatment decreased the risk of developing vascular complications by 57% as compared to HDL-C < 40 mg/dl. The risk of vascular complications was 3.05 times higher among patients with baseline LDL-C > 100 mg/dl as compared to LDL-C ≤ 100 mg/dl. Triglyceride level > 150 mg/dl at the start of anti-diabetic treatment increased the risk of vascular complications by 2.74 times as compared to LDL-C ≤ 150 mg/dl.

**Table 2:** Multivariable analysis using the Gompertz Cox-Regression model for predictor’s vascular complication among type 2 DM patients in university of Gondar referral hospital September, 2005 – March 2017

Variable	Crud HR (95% CI)	Adjusted HR (95% CI)
Age(year)	1.04(1.03, 1.06)	1.02 (0.99, 1.04)
Sex		
Female	1	1
Male	0.47(0.31, 0.73)	0.50(0.27, 0.94)*
Residence		
Rural	1	1
Urban	1.47(0.84, 2.60)	0.51(0.25, 1.02)
Occupation		
Unemployed	1	1
Government	0.80(0.41,1.57)	0.796(0.41, 1.56)
NGO	0.38(0.14,1.05)	0.85 (0.27, 2.70)
Private	0.52(0.31,0.88)	0.83 (0.39, 1.74)
Family history		
Yes	1	1
NO	2.17(1.45, 3.23)	1.25 (0.78, 2.01)
Treatment Type		
OHA	1	1
Insulin	0.79(0.45, 1.36)	0.50(0.28, 1.01)
Insulin + OHA	0.87(0.46, 1.67)	0.91(0.48, 1.72)
BMI kg/m^2^		
18.5–24.99	1	1
< 18.5	0.82(0.32, 2.10)	1.07(0.34, 3.27)
25–29.9	2.04(1.27, 3.29)	0.66(0.37, 1.16)
≥ 30	4.22(2.47, 7.21)	0.84(0.44, 1.61)
Hypertension		
No	1	1
Yes	10.57(5.48, 20.38)	3.99(1.87, 8.56)***
SBP(mm Hg)	1.03(1.0, 1.04)	0.995(0.97, 1.01)
DBP(mm Hg)	1.07(1.05, 1.09)	1.02(0.99, 1.05)
HDL-C(mg/dl)		
< 40	1	1
≥ 40	0.12(0.07, 0.18)	0.43(0.24, 0.77)**
LDL-C(mg/dl)		
≤ 100	1	1
> 100	13.12(7.14,24.10)	3.05(1.47, 6.35)**
Cholesterol (mg/dl)		
≤ 200	1	1
> 200	4.67(2.99, 7.28)	0.76(0.43, 1.36)
Triglyceride(mg/dl)		
≤ 150	1	1
> 150	8.08(4.50, 14.49)	2.74(1.28, 5.84)**
FBS(mg/dl)	1.008(1.006,1.010)	1.00(0.999,1.005)
Creatinine(mg/dl)	1.003(0.995,1.010)	100(0.995, 1.009)
Protein urea		
Negative	1	1
Positive	4.14(2.77, 6.19)	1.69(1.03, 2.78)*

*** expressed as *p*-value< 0.001, **p-value< 0.01, *p-value< 0.05. *BMI* body mass index, *DBP* diastolic blood pressure, *FBS* fasting blood sugar, *HDL-C* high density lipoprotein cholesterol, *LDL-C* low density lipoprotein cholesterol, *SBP* systolic blood pressure

## Discussion

This study mainly investigated the incidence and predictors of vascular complications among type 2 DM patients at University of Gondar Referral Hospital, Ethiopia. This study assessed socio-demographic, clinical and physiologic characteristics of the patients based on the records taken from their medical follow up chart. As a result, factors such as male sex, history of hypertension at baseline, positive protein urea, HDL-C level ≥ 40 mg/dl, LDL-C level > 100 mg/dl and triglyceride > 150 mg/dl were found to be significantly associated with vascular complications.

The cumulative incidence of vascular complications during the study period after a median follow up time of 6.8 years were 28%. This result was slightly less than the study done in Taiwan [28] which showed the incidence to be 30.7% after a median follow up time of 5 years. In our study the incidence rate of vascular complications was 40.6 cases per 1000 person year observation. From this the incidence of coronary heart disease (CHD) and stroke was found to be 16.7 and 17.0 cases per 100 person year observation, respectively. This is lower than a study done in India [29] which showed the incidence rates to be 216 and 115 cases per 1000 person year observation, respectively. In our study the incidence rate of retinopathy was 18.4 cases per 100 person year observation, which is lower than another study done in Kenya [30] which showed the incidence to be 224.7 cases per 1000 person year observation. This could be due to the difference in median follow-up time used by the studies. Because the study in India used longer duration of follow-up (13 years). In addition, it could be due to the age difference of the study participants in which the study in Kenya mainly used patients who were above the age of 50 years. Moreover, the difference could be due to the difference in diagnostic methods used by the studies. In contrast to this, the incidence of retinopathy (18.4), nephropathy(14.4), neuropathy(18.9) and PAD(15.1) cases per 100 person year observation were found to be higher than incidence of retinopathy, nephropathy, neuropathy and PAD to be 78, 58, 13.9, 2, cases per 1000 person year observation in India [31]. This could be due to having a short follow up time (5.7 years) used in the India study.

In this study male type 2 DM patients accounted only 29.9% of the events and were found to have lower risk of developing vascular complications than female patients. This is in line with studies done in Ethiopia [14], India [13] and a met analysis [32] which showed female patients to have higher risk to develop vascular complications. This could be due to the hormonal differences. Because female patients encounter hormonal imbalances and decreased estrogen levels at menopause and at the same time, they lose the vasodilatory and anti-inflammatory activity of estrogen which would lead to endothelial dysfunction [33]. Another reason could be due to sex specific factors like polycystic ovarian syndrome, preeclampsia and gestational DM [34]. Another possible reason may be that women do not engage in as much physical activities as men do; physical activity contributes to improved insulin sensitivity as well as to decreased blood glucose levels and body weight [35]. In contrast to our results other retrospective follow up studies done in Iran [15] and Japan [16] showed males to be at a higher risk of developing vascular complications. Therefore, further research is needed to determine if this sex difference contributes to better outcomes in men with diabetes.

This study’s findings indicated that type 2 DM patients who have history of hypertension at base line had an increased risk of developing vascular complications. This result is consistent with other studies done in Iran [15], Japan [16], India [17], and Ireland [36] which showed that a history of hypertension puts the patients at a higher risk for macro and micro vascular complications. Other studies in Cameroon and Morocco investigated the association between hypertension and specific complications; in this regard type 2 DM patients with hypertension were at increased risk of nephropathy and cardio vascular events [18, 19]. The possible reason could be the effect of hypertension on endothelial cell structure and function that leads to enhanced growth and vasoconstriction; these changes to the endothelium have a key role in the development of arthrosclerosis and glomerulosclerosis which ultimately predisposes patients to vascular complications [37].

In this study, elevated triglyceride level > 150 mg/dl and LDL-C level > 100 mg/dl were found to increase the risk of vascular complications; however an HDL-C level ≥ 40 mg/dl was associated with a decreased risk of vascular complications. This result was in accordance with other studies done in India [20], Singapore [22], Zimbabwe [38] and multi-centered study involving 28 countries from Asia, Africa, Europe and South America [21]. These four studies showed that patients with higher levels of LDL- C to have higher risk to develop vascular complications but patients with the higher levels of HDL-C have a decreased risk. Our result are also consistent with another study in India which showed increased levels of triglycerides to increase the risk of developing vascular complications like stroke and CHD [29]. This could be due to their function since the function of HDL-C is to transport fats (lipids) away from the arterial wall and in to the liver. This eventually reduces risk of accumulation fats and arthroscleroses within the arterial wall and it protects the inner wall of the arteries from damage thereby reducing the risk of CHD, stroke and other vascular diseases [39]. The reverse is true for LDL-C because it transports fates (lipids) to the arteries which in turn produce arthrosclerosis in the arteries of which increases the risk of vascular complications [40]. Excess level triglycerides above the normal range (> 150 mg/dl) also produces plaque in the arteries so it increases the risk of vascular complications. Furthermore, this retrospective follow up study found that patients with positive protein urea have an increased risk of having vascular complications which might be due to the fact that protein urea is an early sign of kidney damage. For this reason, patients with a positive protein urea are at an increased risk of vascular complications like nephropathy in the long run [41].

The clinical importance of this study was to provide information for health professionals and patients about factors that are associated with the risk of vascular complications, as well as, to improve the efforts on prevention. The public health importance of this study is to prevent economic loss associated with these diseases and its complications.

The limitation of this study was the use of secondary data collected retrospectively which results in incompleteness. This study assumed that all the vascular complications are caused by diabetes mellitus and considered vascular complication as a composite outcome for stroke, coronary heart disease, peripheral arterial disease, retinopathy, nephropathy, and neuropathy. This may over estimate the rate of vascular complication.

## Conclusion

In this retrospective follow up study, findings showed that the incidence of vascular complications among type 2 DM patients at University of Gondar Referral Hospital remains a significant public health problem. Hypertension at baseline, LDL-C > 100 mg/dl, triglyceride > 150 mg/dl, HDL-C ≥ 40 mg/dl and male sex were significant predictors of vascular complications among type 2 DM patients. In light of these findings, health professionals in the DM follow up clinics should give targeted intervention for type 2 DM patients with hypertension comorbidity, dyslipidemia and positive protein urea. Patients with hypertension comorbidity should strictly control their hypertension like that of the DM.

## Abbreviations

AHR: Adjusted hazard ratio
AIC: Akaike’s information criteria
BIC: Bayesian information criteria
BP: Blood pressure
CHD: Coronary heart diseases
CHR: Crude hazard ratio
CKD: Chronic kidney diseases
CT: Computerized tomography (CT)
CVD: Cardiovascular diseases
DBP: Diastolic blood pressure
DM: Diabetes mellitus
ECG: Electrocardiogram
FBS: Fasting blood sugar
HDL-C: High density lipoprotein cholesterol
HR: Hazard Raito
HTN: Hypertension
IQR: Inter quartile range
LDL-C: Low density lipoprotein cholesterol
NCEP: National cholesterol education program
PAD: Peripheral arterial disease
PH: Proportional hazard
WHO: World health organization
